# Oxidative Stress Management in Cardiorenal Diseases: Focus on Novel Antidiabetic Agents, Finerenone, and Melatonin

**DOI:** 10.3390/life12101663

**Published:** 2022-10-20

**Authors:** Panagiotis Theofilis, Aikaterini Vordoni, Rigas G. Kalaitzidis

**Affiliations:** Center for Nephrology “G. Papadakis”, General Hospital of Nikaia-Piraeus Agios Panteleimon, 18454 Piraeus, Greece

**Keywords:** oxidative stress, cardiovascular disease, chronic kidney disease, sglt2 inhibitors, glp1 receptor agonists, finerenone, melatonin

## Abstract

Oxidative stress is characterized by excessive production of reactive oxygen species together with exhausted antioxidant defenses. This constitutes a main pathophysiologic process that is implicated in cardiovascular and renal diseases. In particular, enhanced oxidative stress may lead to low-density lipoprotein accumulation and oxidation, endothelial cell activation, adhesion molecule overexpression, macrophage activation, and foam cell formation, promoting the development and progression of atherosclerosis. The deleterious kidney effects of oxidative stress are numerous, including podocytopathy, mesangial enlargement, renal hypertrophy, tubulointerstitial fibrosis, and glomerulosclerosis. The prominent role of oxidative mechanisms in cardiorenal diseases may be counteracted by recently developed pharmacotherapies such as novel antidiabetic agents and finerenone. These agents have demonstrated significant antioxidant activity in preclinical and clinical studies. Moreover, the use of melatonin as a treatment in this field has been experimentally investigated, with large-scale clinical studies being awaited. Finally, clinical implications and future directions in this field are presented.

## 1. Introduction

**Figure 1:**
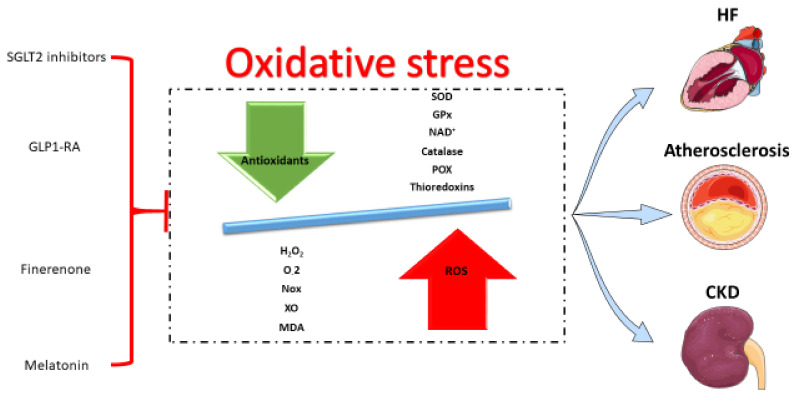
Agents with antioxidant effects, such as sodium-glucose cotransporter-2 (SGLT2) inhibitors, glucagon-like peptide-1 receptor agonists (GLP1-RA), finerenone, and melatonin may induce reductions in reactive oxygen species (ROS) and reinforce antioxidant system activity. Ultimately, the burden of diseases associated with oxidative stress such as heart failure (HF), atherosclerosis, and chronic kidney disease (CKD) is diminished. SOD: superoxide dismutase, GPx: glutathione peroxidase, NAD+: nicotinamide adenine dinucleotide, POX: paraoxonases, Nox: NADPH oxidase, XO: xanthine oxidase, MDA: malondialdehyde.

Cardiovascular and renal diseases are leading causes of morbidity and mortality worldwide. The epidemiological trends of these non-communicable diseases are worrisome, with an increasing incidence and prevalence, especially in developing countries [1,2,3]. Moreover, owing to shared risk factors, these entities frequently coexist. Their pathophysiology is complex, comprising multiple pathways. Among them, oxidative stress is crucial in the development of atherosclerotic diseases, heart failure (HF), and chronic kidney disease (CKD) (Figure 1). Pharmacotherapies with pleiotropic effects, such as sodium-glucose cotransporter-2 (SGLT2) inhibitors, glucagon-like peptide-1 receptor agonists (GLP1-RA), and finerenone have recently been found to reduce the burden associated with these pathologic states. In this narrative review, we discuss the role of oxidative stress in cardiovascular diseases and CKD. Moreover, we present the available preclinical and clinical evidence regarding the antioxidant potential of agents with pleiotropic effects, such as SGLT2 inhibitors, GLP1-RA, and finerenone in cardiac and renal pathologies. Finally, we discuss the importance of melatonin (MT), an endogenous hormone acting mainly through antioxidant mechanisms to ameliorate cardiorenal diseases, according to a plethora of preclinical evidence.

## 2. Oxidative Stress in Cardiorenal Diseases

In live cells, during regular metabolic activities, reactive chemicals such as reactive oxygen species (ROS) and reactive nitrogen species are constantly produced by oxidation reactions, being either enzymatic or nonenzymatic. Free or primary radicals are independent chemical entities containing one or more unpaired electrons. These radicals are extremely reactive while looking for another unpaired electron. A new nonradical molecule is created when two unpaired electrons interact to form a covalent link; but more frequently, when free radicals interact with nonradical molecules, they produce new (secondary) radical molecules that start the chain reaction. Multiple tissues and organs eventually sustain oxidative damage as a result of this main and secondary radical chain reaction.

Numerous ROS-generating systems exist and are associated with the development and progression of cardiorenal diseases. To begin with, NADPH oxidases are among the main representatives. Being present in infiltrating macrophages and vascular wall cells, they comprise two membrane-bound subunits (p22phox and a Nox homolog) and a few cytosolic regulatory subunits [4]. They act by producing superoxide (or hydrogen peroxide in the case of Nox4) from molecular oxygen using NADPH as the electron donor. Experimental evidence in mice suggests the presence of three Nox isoforms in vascular smooth muscle cells (Nox1, Nox4) and endothelial cells (Nox2, Nox4) [5,6,7,8]. The role of Nox in atherosclerosis is diverse, with Nox1 and Nox2 being proatherogenic [9,10,11], while Nox4 is antiatherogenic [12,13,14]. NAPDH may be considered an orchestrator of oxidative stress as it can promote endothelial nitric oxide synthase (eNOS) uncoupling, xanthine oxidase (XO) activity, and mitochondrial ROS production. Moving to XO, its prooxidant effect is based on the generation of superoxide and hydrogen peroxide by using molecular oxygen as an electron acceptor. Among the known triggers of XO production are angiotensin II and oscillatory shear stress [15,16]. XO ultimately adheres to endothelial cells through endothelial glycosaminoglycans [17]. The XO-formed superoxide may be responsible for endothelial cell dysfunction, the initial step of atherosclerosis [17]. Next, mitochondria are responsible for generating physiological levels of superoxide through oxidative phosphorylation. This is subsequently converted to hydrogen peroxide and, ultimately, to water. However, mitochondrial oxidative stress may occur due to the upregulated ROS production and impaired ROS degradation [18]. Finally, eNOS uncoupling is prevalent in cases of oxidative stress, leading to superoxide and peroxynitrite production instead of NO [19]. Those oxidative substances may further augment eNOS uncoupling through tetrahydrobiopterin inactivation, which is a known eNOS cofactor [19]. NO is a crucial mediator of cellular homeostasis as it can regulate vascular tone, prevent platelet activation and aggregation, attenuate leukocyte migration and adhesion, and inhibit vascular smooth muscle cell proliferation [19].

This protective mechanism functions by repeatedly preventing the initial formation of scavenging oxidants and free radicals. Through this, oxidants are changed into less hazardous chemicals, and the secondary generation of harmful metabolites is prevented. The defense system then seeks to fix the molecular damage or strengthen the body’s natural antioxidant defenses, which are made up of nonenzymatic and enzymatic antioxidants. The presence of exhausted antioxidant mechanisms is another point of concern when discussing the effect of oxidative stress on the cardiovascular and renal systems. Among the well-studied antioxidants are superoxide dismutase (SOD), catalase, glutathione peroxidase (GPx), nicotinamide adenine dinucleotide (NAD+), glutathione (GSH), paraoxonases (POX), and thioredoxins. It should be noted that although their upregulated expression induces anti-atherosclerotic effects, extreme overexpression of these antioxidant molecules may lead to proatherogenic effects, as in the case of SOD [20]. Certain polymorphisms in the genes encoding SOD1 (*rs9974610*, *rs10432782*, *rs1041740*) [21], SOD2 (*rs4880*) [22,23,24], SOD3 (*rs1799895*, *rs7655372*) [25,26,27], and GPx1 (*rs1050450*) [26,28,29,30] may also be responsible for atherosclerotic manifestations. Polymorphisms in the genes encoding antioxidant enzymes have been implicated in the development of nephropathy such as with SOD1 (*rs17880135*, *rs202446*, *rs9974610*, *rs204732*, *rs17880135*, *rs17881180*, *rs1041740*) [31,32], SOD2 (*rs4880*, *rs2758329*, *rs8031)* [33,34,35], and GPx1 (*rs1050450*) [33].

Cardiovascular and renal risk factors, namely, diabetes mellitus, arterial hypertension, smoking, and dyslipidemia, are known inducers of augmented oxidative stress. As a result, a series of deleterious sequelae that are associated with atherosclerosis, cardiac and renal dysfunction occur. Starting with atherosclerosis, oxidative stress may initially promote LDL uptake in the vessel wall, possibly due to impaired NO bioavailability [36]. Moving to oxidized LDL (oxLDL), oxidized phospholipids play a major role in mediating many of its proatherogenic traits. Lipid peroxidation can happen by enzymatic or nonenzymatic processes, such as ROS produced by NADPH oxidase or uncoupled eNOS, or by myeloperoxidases, lipoxygenases, cyclooxygenases, and cytochrome P450 [37]. Malondialdehyde, 4-hydroxynonenal, phosphocholine of oxidized phospholipid, and 2-(-carboxyethyl) pyrrole are among the extremely reactive byproducts of lipid peroxidation. They promote the production of structural neoepitopes known as oxidation-specific epitopes (OSEs) [38]. On the surface of apoptotic cells and oxLDL molecules, OSEs have been identified, including oxidized phospholipids and amino groups changed by malondialdehyde. OSEs are recognized by receptors (toll-like receptors (TLRs), scavenger receptors) on endothelial cells and macrophages [38]. This is important for human physiology since tissue homeostasis is maintained by removing dying cells, cellular debris, and damaged molecules [38]. However, when produced in excess, chronic inflammation through proinflammatory molecule secretion is promoted. Sensing of OSEs by endothelial cells leads to oxLDL uptake by lectin-like oxidized LDL receptor-1 (LOX1), TLR2, and TLR4 [38]. As a result, a reduced NO biosynthesis, smooth muscle cell proliferation, and upregulation of adhesion and prothrombotic molecules are noted. Other than OSE overexpression, ROS may mediate shear stress-induced adhesion molecule overexpression [39]. ROS also contribute to macrophage activation and foam cell formation through OSE generation and binding to scavenger receptors or LOX1 [38,40].

In the context of CKD, cellular oxidative stress is an important contributing factor by causing apoptosis, senescence, decreased cell regeneration, and fibrosis in the kidney cells. Extracellular matrix protein buildup, podocyte destruction, mesangial enlargement, renal hypertrophy, endothelial dysfunction, tubulointerstitial fibrosis, and glomerulosclerosis are all effects of oxidative stress [41]. Thus, the decline in renal function and the course of the disease are both further impacted by oxidative stress. Moreover, mitochondrial dysregulation in CKD patients leads to excessive ROS production as a result of abnormal oxidative phosphorylation, and intensifies oxidative stress. Patients with CKD have been discovered to have elevated levels of many oxidative phosphorylation-related genes [42]. Other enzymes that start the creation of ROS, such as Nox, XO, and lipoxygenases, are increased in CKD [43]. XO activity is also higher in CKD, while NO bioavailability is diminished [44,45]. As a result, the increased vascular resistance in renal arteries may promote hypertensive nephropathy [45]. Furthermore, CKD-induced vitamin D deficiency may also promote oxidative stress and aid the progression of CKD [46].

Importantly, chronic inflammation in CKD is mostly attributed to oxidative stress. It has been suggested that chronic low-grade inflammation contributes to the pathogenesis of CKD. Inflammation induced by kidney damage attracts leucocytes and macrophages, resulting in ROS overproduction. Accumulation of ROS engages macrophages and releases cytokines, chemokines, and eicosanoids, which in turn sets off a series of inflammatory responses. The modulation of glomerular filtration rate, renal blood flow, and sodium excretion by cytokines and inflammatory mediators like tumor necrosis factor (TNF), transforming growth factor, and interleukins (ILs) represents a downstream effect [47]. Nuclear factor (NF)-κB, a transcription factor that controls the expression of genes for inflammatory mediators, is also activated by oxidative stress [48]. I-κB, an inhibitory protein that keeps NF-κB in an inactive state, is phosphorylated and degraded by oxidative stress, which causes NF-κB to become active. Antioxidants prevent ROS from activating the NF-κB pathway [49]. The relationship between inflammation and oxidative stress in disease pathogenesis is supported by the high levels of inflammatory markers present in patients with advanced-stage CKD, including C-reactive protein, TNF-α, and IL-6, as well as oxidative stress markers, such as plasma protein carbonyls and F2-isoprostanes [50,51].

## 3. Antioxidant Pharmacotherapies in Cardiorenal Diseases

Many agents have been found with putative antioxidant effects in cardiorenal diseases. However, in this review, we will summarize the evidence on recently established, efficacious cardiorenal pharmacotherapies such as SGLT2 inhibitors, GLP1 receptor agonists, and finerenone. At the same time, we touch upon MT, a well-known endogenous substance which is recently being investigated preclinically in cardiorenal disease. Due to its antioxidant potential, it may end up being a useful addition against cardiorenal diseases’ pathophysiology. Crucially, although these agents act through different pathways, they have all been found to decrease ROS formation and NOX, while enhancing the antioxidant defenses, as discussed below.

### 3.1. SGLT2 Inhibitors

SGLT2 inhibitors have been initially introduced for the treatment of type 2 diabetes mellitus, by inhibiting the reabsorption of glucose in the proximal convoluted tubule. In the trials aimed to establish their cardiovascular safety, these agents were proven cardioprotective by significantly reducing HF hospitalizations, among others. Subsequent trials such as CREDENCE [52], DAPA-CKD [53], and SCORED [54] have documented their efficacy in CKD. Importantly, those findings were also validated in individuals without type 2 diabetes mellitus. Their evaluation in HF populations in the EMPEROR-REDUCED [55] and DAPA-HF [56] trials led to the inclusion of this drug category as a main therapy in the treatment of HF with a reduced left ventricular ejection fraction [57]. As we have shown, SGLT2 inhibitors can lead to the improvement of imaging indices of both systolic and diastolic cardiac function [58]. Lately, the results of SOLOIST-WHF [59], EMPEROR-PRESERVED [60], and DELIVER trials [61] made them the only drug class with positive results in HF with mildly reduced or preserved ejection fraction. Other than cardiorenal protection, these agents may improve fatty liver disease [62].

It is obvious that their mechanism of action is pleiotropic and is independent of the modest glucose-lowering effect. Among the putative mechanisms of action is the restoration of autophagy, the reduction of inflammation, the prevention of endothelial dysfunction, and the downregulation of fibrotic and apoptotic pathways [63,64]. At the level of the kidney, specifically, SGLT2 inhibitors may induce a lowering of intraglomerular pressure, prevent podocytopathy, interact with the sympathetic nervous system, and lower arterial blood pressure. Several reports of recent experimental studies have presented an antioxidant effect of these agents in vitro and in vivo. A reduction of renal ROS, together with upregulation of renal antioxidant mechanisms, have been noted in experimental models of diabetes mellitus, H_2_O_2_-induced renal injury, ischemia-reperfusion injury, and inflammation (Table 1). These observations further support the notion that the effect of SGLT2 inhibitors is not based on glucose lowering. Moreover, cardiac ROS production and antioxidant systems may be modulated through the use of SGLT2 inhibitors. Again, these effects were irrespective of the experimental disease model, being present in diabetic cardiomyopathy, doxorubicin-induced cardiac injury, isoproterenol-induced cardiomyopathy, cardiac ischemia-reperfusion injury, and models of HF (Table 2). An antioxidant effect has also been detected in human umbilical vein endothelial cells and human coronary artery endothelial cells that were stimulated by tumor necrosis factor-α [65].

Scarce clinical evidence exists regarding the antioxidant potential of SGLT2 inhibitors. Lambadiari et al. were the first to report the effect of SGLT2 inhibitors on markers of oxidative stress (thiobarbituric acid reactive substances, malondialdehyde, reducing power, 2,2¢-azino-bis-(3-ethylbenzthiazoline-6-sulphonic acid) radical, and total antioxidant capacity) in 160 participants randomized to SGLT2 inhibitors, GLP1-RA, their combination, or insulin [88]. Individuals on SGLT2 inhibitors alone exhibited only decreases in 2,2¢-azino-bis-(3-ethylbenzthiazoline-6-sulphonic acid) radical after 12 months of follow-up, with no major differences being noted in the rest of the examined markers [88]. In another pilot study, SGLT2 inhibitors induced a reduction in urinary SOD and MnSOD activity, as well as in total antioxidant capacity, in individuals with type 2 diabetes mellitus [89]. The addition of SGLT2 inhibitors to angiotensin-converting enzyme inhibitor ramipril resulted in the lowering of urinary 8-isoprostane concentration compared to the addition of placebo to ramipril in the randomized, double-blind, placebo-controlled, crossover trial of Lytvyn et al. [90]. Moving to a study of 14 non-albuminuric patients with diabetes mellitus, no differences in the urinary oxidative stress marker, 8-hydroxydeoxyguanosine, were noted during the 1-, 3-, and 6-month follow-up [91]. Last but not least, in a circulating proteomics analysis of 1134 patients included in the EMPEROR-PRESERVED and EMPEROR-REDUCED trials, a differential expression of proteins associated with oxidative stress (angiopoietin-related protein 4, insulin-like growth factor-binding protein 4) was documented [92].

### 3.2. GLP1 Receptor Agonists

GLP1-RA constitute an antidiabetic drug category which has been extensively studied in cardiorenal medicine. Their efficacy is mostly centered around atherosclerotic complications prevention, namely, ischemic stroke, as documented in a recent meta-analysis of 6 double-blind, randomized placebo-control trials with 52821 type 2 diabetes mellitus patients [93]. When compared with SGLT2 inhibitors, GLP1-RA were also associated with a lower risk of major adverse limb events within the first two years after initiation [94]. However, there was no difference between those two drug classes in major adverse cardiovascular events in the study of Fu et al. [95], with the exception of a lower risk of ischemic stroke. GLP1-RA may additionally offer renal protection according to meta-analytic evidence [96]. However, their ability to lower the rates of HF hospitalizations is of lesser magnitude compared to SGLT2 inhibitors [97].

Although being solely used in patients with diabetes mellitus, these agents possess several pleiotropic properties. Focusing on their antioxidant effect (Table 3), liraglutide administration in male 129SV mice with streptozocin-induced diabetes promoted an increase in antioxidant molecules (catalase, GPx) in kidney specimens [98]. Low-dose lixisenatide reduced renal malondialdehyde and total Nox, paired with an increase in total antioxidant capacity [99]. This ultimately prevented early diabetic nephropathy development in diabetic Wistar rats [99]. GLP1-RA could also prevent podocyte apoptosis partially through a reduction in oxidative stress [100,101]. Moving to cardiac antioxidant effects, a recent study has shown that liraglutide administration in diabetic Sprague–Dawley rats attenuated the expression of Nox2 in atrial and ventricular tissue compared to placebo [102]. The antioxidant effect of liraglutide in a similar experimental model has been replicated on top of an anti-inflammatory action, leading to diminished cardiac injury [83]. Liraglutide also prevented high glucose-induced neonatal cardiomyocyte apoptosis through the downregulation of malondialdehyde and upregulation of antioxidant SOD [103]. In vitro evidence from H9C2 cardiomyoblasts treated with H_2_O_2_ suggests an increase in antioxidant potential (GPx, catalase, heme oxygenase-1) after administration of a GLP1 analog [104]. Antioxidant effects have been observed in endothelial cells cultured under high glucose and inflammatory settings that were treated with GLP1 analogs or GLP1-RA [105,106,107]. Dual glucagon-like peptide-1/glucose-dependent insulinotropic polypeptide receptor agonists deserve an honorable mention due to their astonishing results in the management of hyperglycemia and obesity, as recently shown [108,109,110,111]. These agents may also act via pleiotropic mechanisms, including attenuation of oxidative stress and inflammation, thus improving diabetes-induced cardiac dysfunction [112].

Although limited data are available from human studies, an antioxidant effect of GLP1-based therapeutics can be suggested. To begin with, Ceriello et al. showed that in both hypoglycemia and hyperglycemia, GLP1 infusion could prevent the increase in the oxidative plasma biomarkers nitrotyrosine and 8-iso prostaglandin F2alpha [119]. Lambadiari et al. showed that 12-month liraglutide administration led to a decrease in malondialdehyde and thiobarbituric acid reactive substances [88]. Liraglutide treatment for 12 weeks in patients with diabetic nephropathy was further associated with a drop in malondialdehyde and an increase in the antioxidant GPx [120]. The antioxidant potential of GLP1-RA, through changes in respective biomarkers, was additionally confirmed in a recently reported systematic review and meta-analysis [121].

### 3.3. Finerenone

Finerenone is a novel, selective, nonsteroidal mineralocorticoid receptor antagonist that has been recently introduced in the treatment of cardiorenal diseases. Landmark trials on patients with diabetic kidney disease have shown an 18% reduction in the incidence of the primary outcome (kidney failure, sustained decrease ≥ 40% in the eGFR, or death from renal causes), 14% reduction in the incidence of the secondary outcome (death from cardiovascular causes, nonfatal myocardial infarction, nonfatal stroke, or hospitalization for HF), and 31% reduction in albuminuria, compared to placebo [122,123]. In patients with HF, finerenone was as efficacious as eplerenone, and may be associated with lesser a lesser increase in potassium levels [124].

Regarding its antioxidant potential (Table 3), an early experimental study by Gueret et al. in mice with coronary artery ligation-induced myocardial infarction initially documented such an effect. Specifically, finerenone abrogated oxidative stress in coronary arteries from noninfarcted mice incubated with low-dose angiotensin-II [125]. At the level of the kidney, finerenone prevented the increase in oxidative stress parameters (malondialdehyde, 8-hydroxy-guanosine) induced by acute bilateral renal ischemia/reperfusion [126]. This was later accompanied by prevention of CKD development (reduced albuminuria, renal vascular resistance, and tubular injury markers) [126]. Finerenone was also found to decrease myocardial ROS production and increase NO bioavailability after short-term administration in Zucker fa/fa rats [113]. This finding may have been responsible for the improvement in diastolic cardiac dysfunction, proteinuria, and tubular injury in the long term [113]. Finally, in Munich Wistar Frömter rats treated with finerenone, aortic ring protein expression of Mn-SOD and Cu/Zn-SOD was enhanced [114]. Total SOD activity was also augmented in the kidneys of those rats treated with finerenone [114]. Collectively, these findings suggest that finerenone’s antioxidant properties may be partly responsible for the cardiorenal benefits seen in randomized clinical trials.

### 3.4. Melatonin

MT is an amphiphilic tryptophan-derived indoleamine that is stimulated in response to darkness. Other than circadian rhythm regulation, MT has potent antioxidant properties, which are exerted either directly or indirectly through binding to its receptors (MT1 and MT2) [127]. Despite being a well-known molecule for decades, its therapeutic potential in the context of cardiorenal diseases is now beginning to be unveiled. Since its receptors are distributed across many organ systems, its beneficial actions may extend to various pathologies. MT has the potential to be an effective therapy for arterial hypertension through modulating endothelial function, oxidative stress, the autonomic nervous system, and the renin-angiotensin system [127]. Additionally, MT may improve beta-cell and insulin sensitivity [127].

By ameliorating those crucial cardiorenal risk factors, along with potent antioxidant properties, MT could emerge as a safe and effective treatment approach in this regard. Numerous preclinical research has been done in this area to investigate the role of antioxidant pathways in mediating the nephroprotective and cardioprotective effects of MT (Table 3). In human renal proximal tubule epithelial cell lines cultured under high glucose conditions, MT improved their antioxidant capacity, evidenced by upregulated catalase and total SOD activity [115]. Transforming growth factor-β1-treated NRK-49F cells treated with MT exhibited lower levels of intracellular ROS and malondialdehyde, as well as ameliorated reductions of the glutathione/oxidized glutathione ratio [128]. Moreover, the addition of MT treatment in diabetic Wistar rats receiving insulin resulted in downregulated expression of GSH, GSH reductase, glucose-6-phosphate dehydrogenase, and GSH-S-transferase in the renal cortex [129]. MT also improved DN in albino rats by suppressing renal malondialdehyde and stimulating antioxidant systems such as GSH, SOD, and catalase [116].

Several studies have also been performed in various experimental models of cardiovascular disease, assessing the antioxidant properties of MT. In rats with doxorubicin- or carbon tetrachloride-induced cardiotoxicity, MT decreased cardiac malondialdehyde [130,131,132]. In hypercholesterolemic mice with air pollution-provoked cardiac dysfunction, MT alleviated mitochondrial oxidative stress by regulating sirtuin 3-mediated SOD2 deacetylation [133]. Moving to H_2_O_2_-induced injury in H9C2 cells, MT abrogated the increases in ROS production by increasing the activity of antioxidant systems (GSH, GPx, SOD), through the mitogen-activated protein kinase/extracellular signal-regulated kinase pathway [117]. Antioxidant effects were reported in Sprague–Dawley rats with myocardial ischemia/reperfusion injury, where MT decreased cardiac malondialdehyde while increasing cardiac SOD and GPx by activating the JAK2/STAT3 signaling pathway [134]. Similar results have been documented in other preclinical studies of cardiac ischemia/reperfusion injury [135,136,137,138]. Activation of cardiac MT2, but not MT1 receptors, may be responsible for this effect [139]. Next, diabetic cardiomyopathy was ameliorated in diabetic Wistar rats treated with MT through an antioxidant response, as shown by Kandemir et al. [118]. Such an effect may be mediated by the 5′ AMP-activated protein kinase/sirtuin 1 pathway [140]. The antioxidant properties of MT have also been observed in experimental models of angiotensin-II-induced cardiac hypertrophy [141], exhaustive exercise-induced cardiac injury [142], and myocardial infarction [143].

Although contemporary research on the cardiorenal benefits of MT is extensive at the preclinical level, strong evidence is limited from clinical studies. Beginning with the renal effects, nocturnal MT 10 mg resulted in a better glycemic profile and oxidative stress indicators in a recently published randomized controlled study of 60 patients with diabetic kidney disease [144]. Similar results were shown in diabetic patients on maintenance hemodialysis, who also had an improvement in inflammatory markers [145]. In a recently published randomized controlled study, kidney transplant recipients were given either MT or a placebo; the MT group showed decreased neutrophil gelatinase-associated lipocalin levels as well as lower levels of inflammatory and oxidative stress indicators [146]. However, no research has been done to determine how MT affects the course of CKD and significant cardiorenal consequences in CKD patients. Examining the function of MT throughout the various CKD phases would be intriguing because CKD is a disorder that causes premature aging. Moving to cardiac effects, in a small scale, placebo-controlled double-blinded randomized clinical trial of 92 patients with HF and a reduced ejection fraction, nighttime MT at a dose of 10 mg decreased the concentration of natriuretic peptides and improved the quality of life of the patients, compared to placebo [147]. Moreover, in recently reported systematic reviews and meta-analyses, MT conferred cardiac protection and improved cardiac function [148,149]. At the same time, it reduced the level of cardiac injury markers, inflammatory cytokines, and oxidative markers while it increased the concentration of antioxidant factors [148]. As with renal studies, evidence is lacking regarding the prevention or prognosis of cardiovascular disease, and future studies are needed in this regard.

## 4. Clinical Implications and Future Directions

Regarding clinical implications, SGLT2 inhibitors have been established in the treatment of cardiorenal diseases due to their overwhelming clinical benefit. However, the burden of side effects, physician inertia, and insurance issues may lead to higher than expected discontinuation rates [150], which should be further assessed in future real-world clinical practice studies. Moreover, the importance of SGLT2 receptor selectivity or dual SGLT1:SGLT2 inhibition deserves further validation, both in its antioxidant potential and its potentially incremental clinical benefit. Head-to-head trials could provide additional evidence in this direction.

Moving to GLP1-RA, another class with proven cardiorenal benefits used solely in patients with type 2 diabetes mellitus, we should state that there may be significant within class variations regarding efficacy, weight loss, and tolerability [151]. While semaglutide may provide the greatest benefit in glycemia management and weight loss, it is accompanied by a high burden of gastrointestinal adverse events, which could result in discontinuation. A lower risk of adverse events could be attributed to exenatide, and lixisenatide, at the loss of efficacy, however. Finally, liraglutide may possess the greatest balance between efficacy and safety. Patient satisfaction should also be taken into account, being higher in cases of once weekly injections compared to twice daily. Although discontinuation rates have been below 10% in clinical trials, this number is believed to be significantly higher in real-life clinical practice. Therefore, selection of the most appropriate agent should be individualized to accommodate cardiorenal benefit (seen with dulaglutide, liraglutide, and injectable semaglutide), glycemia management, weight loss, and tolerability in the most optimal way.

Finerenone, although beneficial in the completed clinical trials, has been examined only in the setting of diabetic CKD. The clinical spectrum of its efficacy may extend to non-diabetic CKD and HF should the ongoing randomized clinical trials (FIND-CKD/NCT05047263 and FINEARTS-HF/NCT04435626) provide positive results. In addition, further preclinical and clinical evidence is needed to determine whether the antioxidant effect is the driving force of its clinical benefit. Finally, despite being a promising therapeutic tool with minimal adverse effects according to the evidence mentioned above, MT’s optimal timing and dosage have not been clarified and represent an existing gap in evidence. Going forward, MT should be tested in adequately designed trials of individuals at high risk for developing cardiorenal diseases such as the elderly (NCT04631341), as well as those with established atherosclerotic disease, HF, and CKD.

Concerning future directions, several agents are in experimental phase. To begin with, activators of endogenous antioxidant systems may be an interesting option, namely, Nrf2 activators. Dimethyl fumarate, an example of this category, led to reduction in myocardial infarct size in animal models of myocardial ischemia/reperfusion injury and to prevention of atherosclerosis in apoE^−/−^ mice [152,153]. This agent may ameliorate nephrotoxicity due to various causes in in vivo animal models [154,155,156,157]. Moving to Nox inhibition, the dual Nox1/4 inhibitor setanaxib (GKT137831) has been investigated in the experimental setting of doxorubicin-, angiotensin II-, and hypertension-induced cardiac remodeling, displaying cardioprotective effects based on antioxidant and antifibrotic action [158,159,160]. At the level of the kidney setanaxib rescued diabetic nephropathy by abrogating glomerular hypertrophy, mesangial matrix expansion, albuminuria, and podocytopathy [161,162,163,164]. Anti-atherosclerotic effects have also been reported [161,165].

Finally, the role of nanoparticles deserves an honorable mention. Loading of nanoparticles with antioxidants that can directly target the area of excessive ROS production is significant, as shown experimentally. H_2_O_2_-responsive nanoparticles have demonstrated efficacy in the preclinical setting of renal and myocardial ischemia/reperfusion injury, by releasing vanillyl alcohol [166,167]. Other antioxidants may also be loaded, such as SOD1, promoting cardioprotective actions [168,169]. Recently, Choi et al. developed nanomicelles able to sense ROS and loaded them with catalase-mimicking 1-dodecanethiol stabilized Mn_3_O_4_. Ultimately, the authors noted that inflammation and apoptosis were attenuated in the renal ischemia/reperfusion injury mouse model [170].

While the reduction of oxidative stress may be beneficial in the setting of cardiorenal pathology, it should be stressed that the non-specific suppression of ROS may account for the lack of benefit observed in clinical studies, since it could disrupt important ROS-mediated cellular signaling. Accordingly, targeted therapies such as nanoparticles may pave the way for selective antioxidant treatment at sites of ROS overexpression, such as the failing heart and kidneys.

## 5. Conclusions

Oxidative stress is a deleterious process that is involved in the pathophysiology of cardiorenal diseases, promoting their development and progression. Recently established pharmacotherapies possess pleiotropic effects that include antioxidant mechanisms, which may partly contribute to the positive effects seen in trials of populations with cardiovascular or renal disease. Research in this field is continuous, and future adequately-sized, randomized studies should further define the importance of these antioxidant effects in the management of cardiorenal diseases.

## Figures and Tables

**Table 1:** Preclinical evidence of renal antioxidant effects of sodium-glucose cotransporter-2 (SGLT2) inhibitors.

Study	Experimental Model	Disease Type	SGLT2 Inhibitor	SGLT2 Inhibitor Effect
Ashrafi Jigheh et al. [66]	Wistar rats	DM	Empagliflozin	↓ renal MDA ↑ renal SOD and GPx
Kimura et al. [67]	OLETF rats	DM	Canagliflozin	↓ renal MDA, 4HNE, Nox2, and Nox4
Das et al. [68]	Proximal tubular epithelial cells	High glucose	Empagliflozin	↓ O_− 2_ and H_2_O_2_ generation
Zaibi et al. [69]	Human proximal tubular cells	H_2_O_2_-induced injury	Dapagliflozin	↓ cytosolic ROS production
Ahmed et al. [70]	Wistar rats	DM post-MI	Empagliflozin	↓ renal Nox2 and Nox4 mRNA
Hudkins et al. [71]	BTBR ob/ob mice	DN	Empagliflozin	↓ urinary markers of RNA/DNA damage ↓ carbonyl oxidation in situ
Ala et al. [72]	Wistar rats	Renal IR injury	Empagliflozin	↓ renal MDA
Malinska et al. [73]	Spontaneously hypertensive rats	Inflammation	Empagliflozin	↑ renal GPx, CAT, GSH ↓ renal CD, TBARS
Ye et al. [74]	C57BL/6J mice	Obesity	Empagliflozin	↑ heme oxygenase-1

DM: diabetes mellitus, MDA: malondialdehyde, SOD: superoxide dismutase, GPx: glutathione peroxidase, OLETF: Otsuka Long-Evans Tokushima Fatty, 4HNE: 4-hydroxynonenal, Nox: nicotinamide adenine dinucleotide phosphate oxidase, ROS: reactive oxygen species, MI: myocardial infarction, DN: diabetic nephropathy, IR: ischemia-reperfusion, CAT: catalase, GSH: glutathione, CD: conjugated dienes, TBARS: thiobarbituric acid reactive substances. ↑ indicates an increase, ↓ indicates a decrease.

**Table 2:** Preclinical evidence of antioxidant effects of sodium-glucose cotransporter-2 (SGLT2) inhibitors on the cardiovascular system.

Study	Experimental Model	Disease Type	SGLT2 Inhibitor	SGLT2 Inhibitor Effect
Xing et al. [75]	Sprague-Dawley rats Cardiac myoblasts H9C2	DM	Dapagliflozin	↓ myocardial MDA, Cu/Zn SOD ↓ cardiomyoblast H_2_O_2_, ↑ cardiomyoblast Cu/Zn-SOD expression and total SOD activity
Hsieh et al. [76]	Cardiac myoblast H9C2	Doxorubicin-induced injury	Dapagliflozin	↑ heme oxygenase-1 and NADPH quinone oxidoreductase ↑ SOD activity
Bugga et al. [77]	Sprague-Dawley rats	DM	Empagliflozin	↓ total cellular and mitochondrial ROS
Li et al. [78]	C57Bl/6J	Pressure Overload-Induced HF	Empagliflozin	↑ heme oxygenase-1, NRF-2, catalase ↓ O_2_-, H_2_O_2_
Tsai et al. [79]	Cardiac myoblasts H9C2 Primary cardiomyocytes	Cardiac IR injury	Dapagliflozin	↓ NADPH activity ↓ ROS formation
Rosa et al. [80]	Wistar rats	DM	Dapagliflozin	↓ lipid hydroperoxide ↑ SOD, GPx
Wang et al. [81]	db/db mice Cardiac myoblasts H9C2	DM	Empagliflozin	↓ cardiac 4HNE and 3-nitrotyrosine ↓ cardiac total cellular and mitochondrial ROS ↓ cardiomyoblast total cellular and mitochondrial ROS
Kolijn et al. [82]	Human cardiomyocytes ZDF rats	HFpEF	Empagliflozin	↓ H_2_O_2_, 3-nitrotyrosine ↑ glutathione
El-Shafey et al. [83]	Sprague-Dawley rats	DM	Dapagliflozin	↓ myocardial MDA ↑ myocardial glutathione, catalase
Li et al. [84]	KK-Ay mice	DM	Empagliflozin	↓ myocardial lipid hydroperoxide, MDA, Nox4 ↑ myocardial GPx, SOD
Wang et al. [85]	Sprague-Dawley rats	Isoproterenol-induced cardiomyopathy	Dapagliflozin	↓ myocardial Nox2, MDA, ROS, NADPH activity,
Yurista et al. [86]	Sprague-Dawley rats	Post-MI HF	Empagliflozin	↓ AOPP, Nox2
Uthman et al. [65]	HUVECs HCAECs	TNFα-induced endothelial dysfunction	Empagliflozin	↓ HUVEC and HCAEC ROS production
Rahadian et al. [87]	ApoE^−/−^ mice	DM	Canagliflozin	↓ aortic Nox2, p22phox ↓ urinary 8-hydroxydeoxyguanosine

MDA: malondialdehyde, SOD: superoxide dismutase, NADPH: nicotinamide adenine dinucleotide phosphate, ROS: reactive oxygen species, HF: heart failure, NRF-2: nuclear factor erythroid 2–related factor 2, GPx: glutathione peroxidase, 4HNE: 4-hydroxynonenal, HFpEF: HF with preserved ejection fraction, MI: myocardial infarction, Nox2: NADPH oxidase-2, HUVEC: human umbilical vein endothelial cell, HCAEC: human coronary artery endothelial cell, TNFα: tumor necrosis factor-α. ↑ indicates an increase, ↓ indicates a decrease.

**Table 3:** Selected preclinical evidence of cardiac and renal antioxidant effects of GLP1 receptor agonists, finerenone, and melatonin.

Study	Experimental Model	Disease Type	Agent	Antioxidant Effect
Liljedahl et al. [98]	129SV mice	DM	Liraglutide	↑ renal catalase, GPx
Abdel-Latif et al. [99]	Wistar rats	DM	Lixisenatide	↓ renal MDA and total Nox ↑ total antioxidant capacity
Baylan et al. [102]	Sprague-Dawley rats	DM	Liraglutide	↓ atrial and ventricular Nox2
Nuamnaichati et al. [104]	H9C2 cells	H_2_O_2_	GLP1 analogue	↑ GPx, catalase, heme oxygenase-1
Zhang et al. [103]	Neonatal cardiomyocyte	DM + inflammation	Liraglutide	↓ MDA ↑ SOD activity
Lachaux et al. [113]	Zucker fa/fa rats	Metabolic syndrome	Finerenone	↓ myocardial ROS ↑ NO bioavailability
González-Blázquez et al. [114]	Munich Wistar Frömter rats	CKD	Finerenone	↑ aortic Mn-SOD and Cu/Zn-SOD ↑ renal total SOD activity
Han et al. [115]	Human renal proximal tubule epithelial cells	DM	Melatonin	↑ catalase and SOD activity
Ebaid et al. [116]	Albino rats	DN	Melatonin	↓ renal MDA ↑ GSH, SOD, catalase
Li et al. [117]	H9C2 cells	H_2_O_2_ toxicity	Melatonin	↑ GSH, GPx, SOD
Kandemir et al. [118]	Wistar rats	DM	Melatonin	↑ cardiac SOD, catalase, GPx

DM: diabetes mellitus, GPx: glutathione peroxidase, MDA: malondialdehyde, Nox: NADPH oxidase, SOD: superoxide dismutase, ROS: reactive oxygen species, NO: nitric oxide CKD: chronic kidney disease, DN: diabetic nephropathy, GSH: glutathione. ↑ indicates an increase, ↓ indicates a decrease.

## Data Availability

Not applicable.
